# A Novel Mutation c.841C>T in COPA Syndrome of an 11-Year-Old Boy: A Case Report and Short Literature Review

**DOI:** 10.3389/fped.2021.773112

**Published:** 2021-11-24

**Authors:** Jingxia Zeng, Jing Hao, Wei Zhou, Zhaoqun Zhou, Hongjun Miao

**Affiliations:** ^1^Department of Emergency Medicine, Children's Hospital of Nanjing Medical University, Nanjing, China; ^2^Nanjing Key Laboratory of Pediatrics, Children's Hospital of Nanjing Medical University, Nanjing, China

**Keywords:** COPA syndrome, gene mutation, lung disease, auto-immunity, children

## Abstract

COPA syndrome is a rare autosomal dominant disorder with auto-immune and auto-inflammatory abnormalities. This disease is caused by mutations of COPα, a protein that functions in the retrograde transport from the Golgi to the ER. Here we report the first COPA case of an 11-year-old boy with c.841C>T, p.R281W mutation. The arginine at position 281 was located in a highly evolutionary-conserved region. Immunosuppressive drugs and corticosteroids might not improve the long-term outcome of COPA patients. For patients with pulmonary disease, polyarthritis and/or kidney disorder, and suspected of COPA, genetic analysis should be conducted promptly for early diagnosis.

## Introduction

COPA syndrome (OMIM 616414), an inheritable autosomal dominant disease first reported in 2015, is characterized by inflammatory arthritis, interstitial lung disease (ILD), and immune complex-mediated renal disorder (1). COPA syndrome has been classified as an auto-inflammatory disorder in the 2017 Update of the Classification of Inborn Errors of Immunity by International Union of Immunological Societies (IUIS) (2). It is caused by mutations in the *COP*α gene, the alpha subunit of COPI that facilitates the retrograde transport from the Golgi to the ER. As an innate immunity ER protein, STING acts as a cargo of the retrograde membrane transport. Having bound to cGAMP, STING is translocated from the ER and into the Golgi, then triggers type I interferon and proinflammatory responses through activating interferon regulatory factor 3 (IRF3) and nuclear factor-kappa B (NF-kB) (3, 4). Surf4, a protein that circulates between the ER/ER-Golgi intermediate compartment/Golgi, binds to STING and α-COP, and mediates the retrograde transport of STING to the ER (4). The *COP*α mutation does not affect the overall expression level of COPα in the cell, but can disrupt protein transport, increase ER stress and activate unfolded protein response, all cooperating to trigger auto-inflammatory and auto-immune responses (5). In general, COPA syndrome is a result of immune dysregulation.

COPA is mostly reported in Caucasians and rarely in Asians. In the latest research, 76% of patients presented with symptoms before 5 years old (1). COPA syndrome is manifested by pulmonary symptoms (like cough and tachypnea), and extrapulmonary symptoms (like joint pain and polyarthritis). Some patients need oxygen supplement, and more common than not, some with diffuse alveolar hemorrhage (DAH) require transfusion treatment, intensive care, or even mechanical ventilation (6). Lung symptoms are not always concurrent with joint/kidney diseases.

To date, treatment regimens with corticosteroids, immunosuppressant and biological agents have been tried to treat COPA. The combination of drugs can induce remission, but a long-term use is not feasible due to possible negative effects. In a previous study, the index case and his son received a bilateral lung transplant (7). Krutzke et al. described a successful treatment with baricitinib in a 15-year-old girl of COPA syndrome (8). Here, we report a novel gene mutation in an 11-year-old Chinese boy with COPA syndrome. The patient's parents provided informed consent, and the Ethics Committee of the Children's Hospital of Nanjing Medical University approved the publication of this case report.

## Case Report

**Figure 1:**
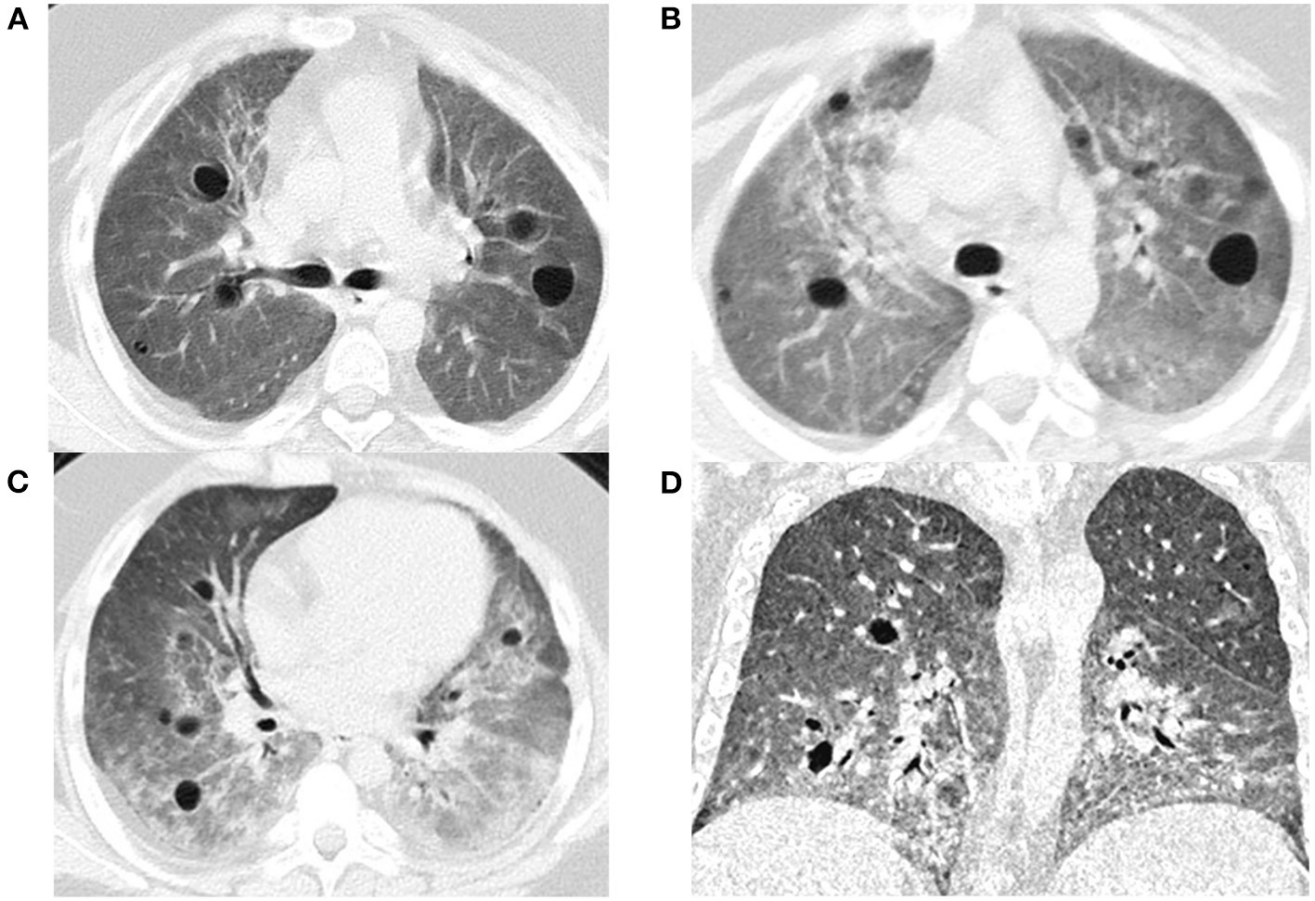
The chest CT image: in lung window, bilateral diffuse ground glass opacities, varying sizes of cyst formation, and bronchial wall thickening in a bronchovascular (**A**: Dec. 2015, **B**: June. 2017, **C**: Nov. 2018, **D**: Mar. 2019).

At the age of 7 years, the case presented his initial symptoms of chronic cough and wheezing, shortness of breath, dyspnea on exertion, mild clubbing and moderate anemia (hemoglobin 61 g/L). He did not have a family history of lung, kidney, or arthritis problems. He was admitted to the Department of Respiratory Medicine at 8-years in December, 2015. Peripheral blood test showed normal C-reactive protein and renal function, with slightly elevated erythrocyte sedimentation rate (ESR 26 mm/h), lactate dehydrogenase (LDH) of 753 U/L, positive antinuclear antibody (Nuclear spot type 1:100); HEP/2 monkey weakly positive, perinuclear anti-neutral cytoplasmic antibody IgG (1:240) and rheumatoid factor (30.4 IU/ml), slightly increased IL-1β (10 pg/ml) and IL-6 (20 pg/ml), and immune dysfunction. His lymphocyte immune function test showed a lymphocyte count of 211/ul: T lymphocytes count of 178/ul (CD3+cells accounting for 84.58%; CD4+cells for 29.82%; CD8+cells for 54.46%; CD4+CD8+cells for 1.32%), NK cell count of 15/ul (7.43%), B lymphocyte count of 4/ul (2.44%); humoral immunity: IgG (7.51 g/L), IgM (0.664 g/L), IgG4 (0.24 g/L), IgA (1.43 g/L), Complement 3 (1.31 g/L), Complement 4 (0.295 g/L). All microbiological results were normal. Echocardiography and joint X-ray also presented normal results. The pulmonary imaging findings included bilateral diffuse ground glass opacities, varying sizes of cyst formation, and bronchial wall thickening on chest computed tomography (CT) (Figures 1A–D). Pathological analysis of video-assisted thoracoscopic lung biopsy suggested the possibility of diffuse pulmonary disease and idiopathic pulmonary hemosiderosis (IPH). Cellular morphology of bronchoalveolar lavage (BAL) revealed follicular bronchiolitis. Pulmonary function test showed forced expiratory volume in 1s (FEV1) 67% and predicted forced vital capacity (FVC) 64%, suggesting mild restrictive ventilation dysfunction.

He was primarily diagnosed with ILD, and thus received corticosteroids therapy from December 22nd, 2015. Intravenous methylprednisolone 80 mg/d (2 mg/kg.d) was administrated for 10 consecutive days, then reduced to 60 mg/d (1.5 mg/kg.d) for another 5 days, followed by oral steroids from January 12th, 2016 (Prednisone 40 mg/d for 20 days and gradually reduced to 15 mg/d in March 14th, 2016). Because the boy received hormone therapy, his weight gained rapidly, fatty liver appeared in the later stage of the disease, and the transaminase increased; therefore, hepatoprotective treatment with glutathione was taken. At the same time, his lung disease progressed rapidly and the wheezing was obvious. He also received nebulization inhalation budesonide suspension to relieve wheezing symptoms. His symptoms were relieved by systemic corticosteroids and the autoantibody turned negative. Considering recurrent chronic cough and shortness of breath, cyclophosphamide impulse treatment was used once a month from April 2017 to June 2017 (0.4g^*^2 days per month, 600 mg/m^2^.d). But cyclophosphamide was discontinued due to a significant increase in liver transaminase. Rituximab was then administered for four times (375 mg/m^2^, once every 3 months). During rituximab treatment, his lung infection aggravated, so a comprehensive antibacterial, antifungal treatment was adopted. Since January 2018, he was treated with oral chloroquine (0.1 g bid, 5 mg/ kg.d) to regulate immunity function, with timely infusion of gamma globulin and albumin.

Owing to high blood sugar, hypertension and disease activity, the patient developed joint pain and spinal compression fracture. His oxygen saturation could not be increased, and the lung disease progressed rapidly. He was hospitalized for four times in the pediatric intensive care unit (PICU). At the later stage, he had difficulty in weaning from mechanical ventilation and all treatments failed. He eventually died of multiple organ failures at the age of 11-year−9-month.

**Figure 2:**
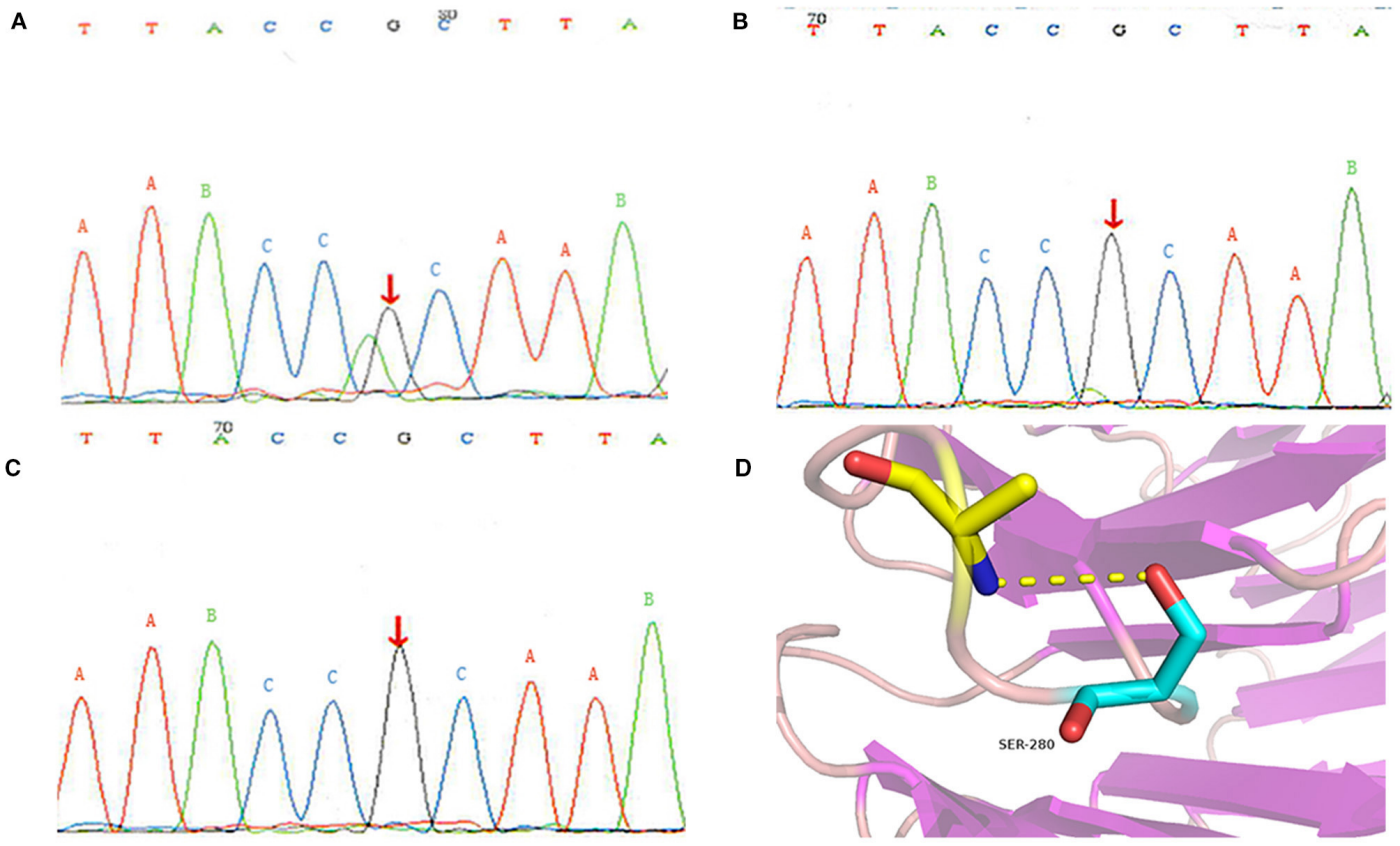
Genetic analysis results: **(A)** heterozygous mutation of COPA gene in the patient; **(B)** there was no mutation in the COPA gene of his father; **(C)** there was no mutation in the COPA gene of his mother; **(D)** Protein conformation after amino acid mutation. The different uppercase letters represent base pairs, arrows A, B and C represent the 841st base, mutation occurs at site A, and no mutation occurs at site B and C.

**Figure 3:**
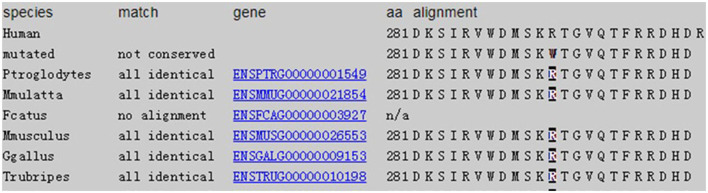
Conservation: amino acids level for non-synonymous changes.

Considering that the patient's lung symptoms could not be reduced by immunosuppressants, whole exome sequencing (WES) was conducted in January 2018 with written informed consent from the patient and his parents. Peripheral blood samples of the patient and his parents were collected and sent to Beijing MyGenostics laboratory. The heterozygous mutation of COPA gene in c.841C>T was detected in exon 9, as evidenced by the mutation of arginine at position 281 into tryptophan (p.R281W) (Figure 2A). The mutation was located between the domains of COPA protein WD6 (241aa-278aa) and WD7 (282aa-319aa). MutationTaster (http://www.mutationtaster.org/), SIFT, polyPHEN2 bioinformatics softwares all predicted that p.R281W mutation is pathogenic. The conservative analysis indicated that the arginine at position 281 was located in a highly evolutionary-conservative region (Figures 2D, 3), suggesting that the c.841C>T (p.R281W) mutation might have dysregulated the function of COPA protein. COPA can be inherited through generations. However, Sanger sequencing verified no heterozygous mutation of c.841C>T (p.R281W) in his parents, confirming the *de novo* mutation of this autosomal dominant (AD) disease (Figures 2B,C).

## Discussion

The lung involvement of autoimmune disorder is rare in children. The pathophysiology of COPA syndrome is not clear yet. Imaging analysis demonstrated the normal distribution of protein in the whole cell in COPA patients (9), indicating that the genetic changes impair the function of COPA protein.

COPA mutation leads to ER stress and the expansion of Th17 cells, then elevates transcript encoding for cytokines IL-1β, IL-6 and IL-23 (10). Increased expression of IL-1β, IL-6 and IL-23 then upregulates Th17 cells and reduces CD4 cells. In our report, the levels of IL-1β and IL-6 increased slightly, and the percentage of CD4 cells decreased, suggesting the presence of inflammatory reactions and autoimmune disorders. The clinical presentations of COPA are similar to those of STING-associated vasculopathy with onset in infancy (SAVI), a type-I interferon dysregulation caused by TMEM173-activated mutations (6, 11). But pulmonary inflammation plays differential roles in COPA and SAVI (11). Recent reports show that a defect in COPI transport causes ligand-independent activation of STING and COPA maintains immune homeostasis by regulating STING transport at the Golgi (3, 4, 12). These findings tell the link between COPA mutants and STING-mediated interferon signaling (3, 4, 12, 13). The expression of COPA mutants enables STING to accumulate in the Golgi, whereas COPA overexpression does not affect the localization of STING in the ER. The loss of COPA function leads to enhanced type I IFN signaling, due to the failure of Golgi-to-ER STING retrieval (13).

All COPA mutants identified are missense single-nucleotide polymorphisms in exons encoding the WD40 conserved domains of the COPA protein. To date, 38 COPA patients have been reported (1, 7, 8, 14–20) (Table 1). So far, the mutated genes found in COPA patients include p.Pro145Ser (18), p.Lys230Asn, p.Arg233His, p.Trp240Arg, p.Glu241Lys, p.Glu241Ala (14), p.Asp243Asn, p.Ala239Pro, p.Asp243Gly (1), p.Val242Ala (19), and p.Val242Gly (20). These mutants are located in the WD40 domain of COPA protein. Most of the children had pulmonary lesions and 95% had manifestations of arthritis (6). Unlike others, all patients carrying the p.Glu241Lys mutation develop no kidney disease (1), which suggests that the mutation does not have deleterious effects on renal function. In our case, the heterozygous mutation of COPA gene in c.841C>T was detected in exon 9, resulting in the change of arginine (R) at position 281 into tryptophan (W). The mutation was located between the domains of COPA protein WD6 and WD7. The pathogenic mutation does not change protein expression level, but can impair protein function. Here, the mutation site we reported leads to serious lung disease, but no renal manifestations.

**Table 1:** The mutated genes found in COPA patients.

**Region**	**Exon**	**Domain**	**cDNA change**	**Amino acids change**	**Mutation**	**Relation**
China	6	WD40	c.433C>T	p.Pro145Ser (p.P145S)	Heterozygous mutation	Pathogenic
USA	8	WD40	c.690G>T	p.Lys230Asn (p.K230N)	-	Pathogenic
USA Italy France	8	WD40	c.698G>A	p.Arg233His (p.R233H)	Missense variant	Pathogenic
-	9	WD40	c.715G>C	p.Ala239Pro (p.A239P)	Spontaneous heterozygous mutation	Likely pathogenic
USA	9	WD40	c.718T>C	p.Trp240Arg (p.W240R)	-	-
Germany	9	WD40	c.719G>C	p.Trp240Ser (p.W240S)	-	-
Germany	9	WD40	c.719G>T	p.Trp240Leu (p.W240L)	-	-
USA, Iceland	9	WD40	c.721G>A	p.Glu241Lys (p.E241K)	Non-synonymous variant	Pathogenic
USA	9	WD40	c.722A>C	p.Glu241Ala (p.E241A)		
China	9	WD40	c.725T>C	p.Val242Ala (p.V242A)	Heterozygous missense mutation	Likely pathogenic
Japan	9	WD40	c.725T>G	p.Val242Gly (p.V242G)	Missense heterozygous variant	Pathogenic
UK	9	WD40	c.727G>A	p.Asp243Asn (p.D243N)	-	-
USA	9	WD40	c.728A>G	p.Asp243Gly (p.D243G)	-	Pathogenic
Germany	9	-	c.841C>T	p.Arg281Trp (p.R281W )	-	-
China^*^	9	between WD6 and WD7	c.841C>T	p.Arg281Trp (p.R281W )	Spontaneous missense mutation	Likely pathogenic

* *Represents the patient in our study. -, The information is unknown*.

Prompt genetic analysis is suggested for the patient showing lung disease and polyarthritis and suspected of COPA. If the patient is positive for COPA syndrome, the aim of treatment is to reduce the risk of pulmonary hemorrhage and severe complications. As an inhibitor of JAK-1 and JAK-2, baricitinib may be an effective drug for COPA syndrome (8). Given the role of ER-Golgi axis in controlling autoinflammation related to COPA syndrome, Type 1 interferons, such as JAK inhibitors or anti-interferon monoclonal antibodies, are expected to exert beneficial effects. STING palmitoylation inhibitors may provide a new treatment option for COPA syndrome in the future (4).

## Conclusions

This is the first report of c.841C>T, p.R281W mutation in a COPA case. The c.841C>T, p.R281W mutation induces serious lung disease, joint pain, and positive autoantibodies, but no kidney diseases. Early diagnosis and prompt management with immunosuppressants are beneficial, but may not improve long-term outcomes.

## Data Availability Statement

The original contributions presented in the study are included in the article/supplementary material, further inquiries can be directed to the corresponding author/s.

## Ethics Statement

The Ethics Committee of Children's Hospital of Nanjing Medical University approved this study (No. 202004032-1). Written informed consent to participate in this study was provided by the participants' legal guardian/next of kin.

## Author Contributions

JZ and JH performed the clinical and laboratory data collection and wrote the manuscript. WZ performed the molecular diagnostics and wrote the manuscript. ZZ and HM contributed the important intellectual content during manuscript drafting and revision. Manuscript revision was performed by all authors. All authors read and approved the final manuscript.

## Conflict of Interest

The authors declare that the research was conducted in the absence of any commercial or financial relationships that could be construed as a potential conflict of interest.

## Publisher's Note

All claims expressed in this article are solely those of the authors and do not necessarily represent those of their affiliated organizations, or those of the publisher, the editors and the reviewers. Any product that may be evaluated in this article, or claim that may be made by its manufacturer, is not guaranteed or endorsed by the publisher.
